# Daily HIV pre-exposure prophylaxis (PrEP) with tenofovir disoproxil fumarate-emtricitabine reduced *Streptococcus* and increased *Erysipelotrichaceae* in rectal microbiota

**DOI:** 10.1038/s41598-018-33524-6

**Published:** 2018-10-12

**Authors:** Michael P. Dubé, Sung Yong Park, Heather Ross, Tanzy M. T. Love, Sheldon R. Morris, Ha Youn Lee

**Affiliations:** 1 Department of Medicine and Division of Infectious Diseases, Keck School of Medicine, University of Southern California, Los Angeles, CA USA; 2 Department of Molecular Microbiology and Immunology, Keck School of Medicine, University of Southern California, Los Angeles, CA USA; 3 Department of Biostatistics and Computational Biology, School of Medicine and Dentistry, University of Rochester, Rochester, NY USA; 4 University of California San Diego Antiviral Research Center, San Diego, CA USA

## Abstract

Daily PrEP is highly effective at preventing HIV-1 acquisition, but risks of long-term tenofovir disoproxil fumarate plus emtricitabine (TDF-FTC) include renal decline and bone mineral density decrease in addition to initial gastrointestinal side effects. We investigated the impact of TDF-FTC on the enteric microbiome using rectal swabs collected from healthy MSM before PrEP initiation and after 48 to 72 weeks of adherent PrEP use. The V4 region of the 16S ribosomal RNA gene sequencing showed that *Streptococcus* was significantly reduced from 12.0% to 1.2% (p = 0.036) and *Erysipelotrichaceae* family was significantly increased from 0.79% to 3.3% (p = 0.028) after 48–72 weeks of daily PrEP. *Catenibacterium mitsuokai*, *Holdemanella biformis* and *Turicibacter sanguinis* were increased within the *Erysipelotrichaceae* family and *Streptococcus agalactiae*, *Streptococcus oralis*, *Streptococcus mitis* were reduced. These changes were not associated with host factors including PrEP duration, age, race, tenofovir diphosphate blood level, any drug use and drug abuse, suggesting that the observed microbiome shifts were likely induced by daily PrEP use. Long-term PrEP resulted in increases of *Catenibacterium mitsuokai* and *Holdemanella biformis*, which have been associated with gut microbiome dysbiosis. Our observations can aid in characterizing PrEP’s side effects, which is likely to improve PrEP adherence, and thus HIV-1 prevention.

## Introduction

Truvada, a fixed-dose combination of the nucleoside reverse transcriptase inhibitors (NRTI) tenofovir disoproxil fumarate (TDF) plus emtricitabine (FTC), was approved for daily HIV-1 pre exposure prophylaxis (PrEP) in 2012, and by 2015 more than 79,000 people in US are estimated to have started TDF-FTC for PrEP^1^. Daily PrEP is highly effective at preventing HIV-1 infection, reducing HIV-1 acquisition by over 90% in MSM^2^. As a key part of the national HIV/AIDS strategy, PrEP use is expected to continue to increase, and thus understanding the side effects of widespread PrEP adoption is an important task.

In randomized studies in long-term PrEP users, kidney function decline^3^ and bone mineral density decrease^4^ have been reported, which are in concordance with the observations among TDF-treated HIV-infected individuals^5,6^. Renal tubular toxicity of TDF has been suggested as a mechanism for kidney abnormalities^7^. TDF-induced bone mineral density decreases have been explained by osteoblast gene expression changes^8^ and tubular dysfunction and resulting phosphaturia^9^. We may gain further insights on these long-term PrEP side effects by profiling the gut microbiome as the human gut microbiota is reported to be correlated with both chronic kidney disease and osteoporosis. Increases in uremic toxins and inflammation among chronic kidney disease patients were in part due to the gut microbiome’s role in the fermentation of short-chain fatty acids^10^. Several studies reported a significant association between the intestinal microbiota, calcium absorption, and bone health^11,12^. As suggested from these findings, study of the microbiome profile, in the absence of HIV infection during PrEP, may provide an important correlate for addressing long-term side effects attributable to TDF-FTC use.

A direct interaction between a drug and the enteric microbiome has been demonstrated for different classes of drugs^13–15^. Other drugs may also have indirect and unintended antimicrobial activities^16^. Indeed, a recent population-level study found medication to have the greatest influence on gut microbiome composition, compare to other covariates such as gender, diet, and health status^17^.

In this study, we investigated the impact of PrEP on the enteric microbiome using serial specimens collected from healthy individuals before initiation of PrEP (baseline) and after 48 to 72 weeks of adherent PrEP use in the California Collaborative Treatment Group study 595 (NCT01761643). CCTG 595 was a randomized controlled trial in high risk MSM and transgender women to compare the impact of daily text messaging to standard care on daily TDF-FTC adherence^18^. We chose study participants with documented adequate adherence to PrEP and these are optimal for investigating the direct impact of daily PrEP on the enteric microbiome profile. By performing next-generation sequencing of the bacterial 16S ribosomal RNA (rRNA) gene, we assessed the relative abundance of each family and genus before and after PrEP administration and identified microbiome shifts after long-term PrEP. In addition, we conducted long-read next-generation sequencing of bacterial 16S-23S rRNA to identify which species are impacted by long-term daily PrEP.

## Results

We compared the microbiome composition of 8 study participants’ rectal specimens before PrEP administration and after 48 to 72 weeks of adherent PrEP use. Table 1 lists each study participant’s age, race, duration of PrEP use, tenofovir diphosphate (TFV-DP) level measured at 48 weeks post PrEP initiation, and any other drug usage and abuse. The next-generation sequencing reads of the V4 region of the 16s ribosomal RNA gene were obtained and processed as described in Methods. The number of sequencing reads before and after processing are provided in Supplementary Table S1.

**Table 1:** Demographic and clinical information of 8 PrEP study participants.

Participant	PrEP Duration	Age	Race	TFV-DP (fmol/punch)	Any drug use	Any drug abuse	Rectal douching
WWS	72	39	Asian	2059	Yes	No	No
XNT	72	28	White	1755	Yes	Yes	Yes
WD5	72	52	White	1620	No	No	Yes
WT9	72	29	White	1447	Yes	Yes	Yes
XRQ	72	33	White	1417	No	No	Yes
7RJ	48	22	Multiple	1163	Yes	Yes	Yes
7FX	60	38	Black	1151	Yes	No	Yes
7NR	48	37	Multiple	996	Yes	No	Decline*

*Declined to answer.

PrEP-induced changes in the microbiome composition were assessed with two methods. First, we statistically addressed the impact of PrEP on the microbiome composition using a permutation test. To control for the small sample size, we performed a permutation test in which we randomly permuted all specimens’ PrEP status (pre-PrEP or post-PrEP) to identify any meaningful differences in the microbiome composition at the family and genus levels. An empirical p-value was obtained for each family or genus through 100,000 PrEP status permutations. We also conducted a paired-permutation test by permuting each individual’s PrEP status and evaluating the chance to observe the measured *t*-statistic, compared to the *t*-statistic distribution for all possible 2^8^ arrangements. Second, we calculated the log fold change of each family or genus, defined as log_2_ of the ratio between mean abundances after and before PrEP.

**Figure 1:**
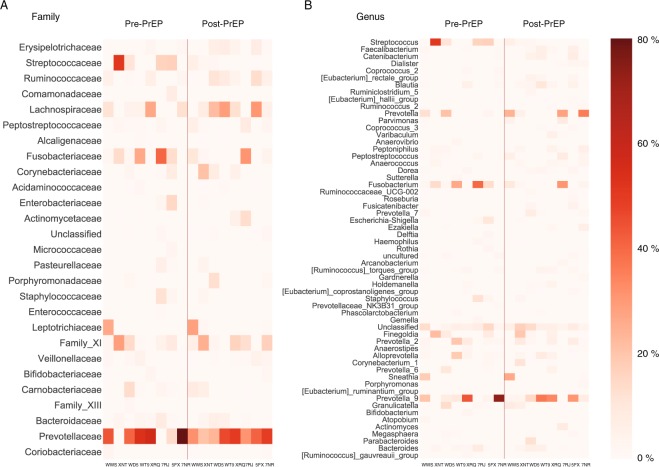
Microbial abundance profiles before and after long-term PrEP with TDF-FTC. (**A**) Each family’s relative abundance within eight CCTG-595 study participants before and after PrEP. The microbiome profile was determined by MiSeq high-throughput sequencing of the V4 region of the 16s ribosomal RNA gene. Families were sorted by the empirical p-value from the permutation test. The level of *Erysipelotrichaceae* significantly increased with 48–72 weeks PrEP administration (p = 0.028). *Streptococcaceae* level significantly decreased after PrEP treatment (p = 0.039). (**B**) Each genus abundance for eight participants before and after PrEP, sorted by the permutation test p-value. The relative abundance of *Streptococcus* significantly decreased after PrEP treatment (permutation test, p = 0.036).

**Figure 2:**
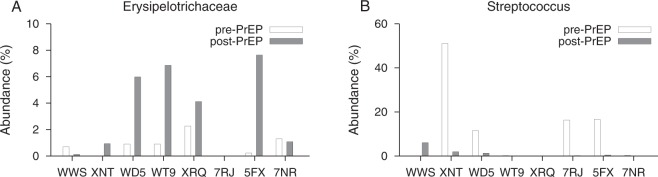
*Erysipelotrichaceae* and *Streptococcus* abundance changes by TDF-FTC. (**A**) The *Erysipelotrichaceae* abundance before and after PrEP for each of eight CCTG 595 study participants who were on daily PrEP for 48–72 weeks. The average abundance of *Erysipelotrichaceae* was increased from 0.79% to 3.3%, resulting in a mean log_2_ (fold change) of 2.1. Both permutation and paired-permutation tests indicate a statistically significant increase in the relative abundance of *Erysipelotrichaceae* after PrEP (p = 0.028 and p =0.031, respectively). (**B**) The *Streptococcus* abundance before and after PrEP for each of eight study participants. On average, the relative abundance of *Streptocuccus* was decreased from 12.0% to 1.2% after a minimum of 48 weeks PrEP treatment (permutation test p = 0.036 and paired-permutation test, p = 0.035). The resulting mean log_2_ (fold change) was −3.3.

Figure 1 plots the abundance of each family and genus from eight subjects before and after PrEP, sorted by the empiric p-value obtained from the permutation test. At the family level, the permutation test indicated that the abundances of *Erysipelotrichaceae* and *Streptococcaceae* were statistically significantly altered with a minimum of 48 weeks PrEP administration (p = 0.028 and p = 0.039, respectively). *Erysipelotrichaceae* and *Streptococcaceae* remained significantly different under the paired-permutation test (p = 0.031 and p = 0.039, respectively). Pre-PrEP specimens had 0.79% of *Erysipelotrichaceae* on average, however, its relative abundance was increased to 3.3% after PrEP, resulting in a mean log_2_(fold change) of 2.1. As shown in Fig. 2A, six out of eight participants showed *Erysipelotrichaceae* increase after PrEP. On the contrary, relative abundance of *Streptococcaceae* was decreased after PrEP with log_2_ (fold change) of −3.3. Four participants showed a considerable reduction of *Streptococcaceae* with an over 10 percent decrease.

At the genus level, *Streptococcus* was significantly reduced after PrEP administration (permutation test, p = 0.036 and paired-permutation test, p = 0.035). The average relative abundance of *Streptococcus* across eight study participants was 12.0% before PrEP initiation. However, after 48–72 weeks of daily TDF-FTC, the average abundance was reduced to 1.2%. Figure 2B compares the *Streptococcus* abundance before and after PrEP for each of eight study participants. The mean log_2_(fold change) over eight participants was −3.3 and we observed more than a 10 percent reduction in *Streptococcus* level in four participants (Fig. 2B). In particular, participant XNT showed a reduction from 51% to 1.9%. Within the *Streptococcaceae* family, Streptococcus was the dominant genus (>99%) in all eight participants, resulting in significant shifts at both the family and genus levels. Table 2 lists each family’s p-value and fold change.

**Table 2:** Abundance difference in given family and genus before and after minimum 48 weeks of PrEP administration.

Family	log_2_ (fold change)	p value	Genus	log_2_ (fold change)	p value
Erysipelotrichaceae	2.08	0.028	Streptococcus	−3.27	0.036
Streptococcaceae	−3.27	0.039	Faecalibacterium	1.77	0.054
Ruminococcaceae	1.43	0.058	Catenibacterium	2.96	0.056
Comamonadaceae	Inf	0.10	Roseburia	1.61	0.061
Lachnospiraceae	0.81	0.16	Dialister	1.77	0.062
Peptostreptococcaceae	1.16	0.17	Eubacterium_rectale_group	1.85	0.081
Alcaligenaceae	−1.24	0.18	Coprococcus_2	−7.28	0.083
Fusobacteriaceae	−1.02	0.18	Blautia	1.49	0.092
Actinomycetaceae	3.54	0.18	Ruminiclostridium_5	2.97	0.096
Acidaminococcaceae	1.00	0.19	Eubacterium_hallii_group	1.15	0.12
Corynebacteriaceae	1.51	0.20	Varibaculum	6.96	0.12
Enterobacteriaceae	−3.66	0.21	Ruminococcus_2	1.63	0.13
Micrococcaceae	Inf	0.24	Arcanobacterium	7.20	0.13
Enterococcaceae	−4.59	0.27	Parvimonas	3.25	0.13
Pasteurellaceae	−0.94	0.27	Haemophilus	−3.52	0.13
Porphyromonadaceae	2.33	0.28	Coprococcus_3	1.35	0.14
Staphylococcaceae	−2.34	0.28	Prevotella	1.23	0.14
Leptotrichiaceae	0.24	0.29	Peptoniphilus	1.14	0.15
Family_XI	0.41	0.30	Dorea	0.78	0.16
Veillonellaceae	0.33	0.35	Peptostreptococcus	1.31	0.16
Bifidobacteriaceae	−0.55	0.36	Anaerovibrio	−3.29	0.16
Carnobacteriaceae	−0.40	0.39	Anaerococcus	1.07	0.16
Family_XIII	−1.42	0.41	Corynebacterium_1	1.51	0.17
Bacteroidaceae	−0.062	0.48	Sutterella	−1.27	0.17
Prevotellaceae	0.022	0.48	Fusobacterium	−1.02	0.17
Coriobacteriaceae	−0.48	0.50	Ruminococcaceae_UCG-002	1.37	0.18

The p value from the permutation test is presented.

**Figure 3:**
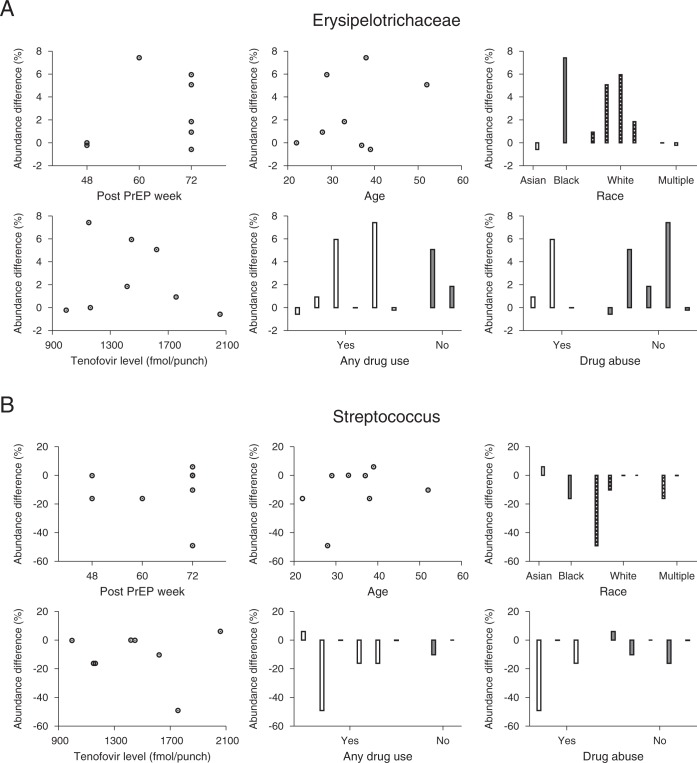
Microbiome changes after TDF-FTC and host factors. (**A**) Eight individuals’ *Erysipelotrichaceae* abundance differences before and after PrEP. The *Erysipelotrichaceae* abundance difference was not dependent on the duration of PrEP (Spearman’s correlation *ρ* = 0.21 and p = 0.62). Age did not significantly correlate with the *Erysipelotrichaceae* abundance change after PrEP (*ρ* = 0.071 and p = 0.88). The *Erysipelotrichaceae* abundance change was not sensitive to race (1 Asian vs. 1 Black vs. 4 Whites vs. 2 Multiples, Kruskal-Wallis test, p = 0.37) and was not correlated with intracellular tenofovir disphosphate level measured at 48 weeks after PrEP initiation (*ρ* = −0.19 and p = 0.66). The *α*-diversity abundance change did not differ between participants with and without any other drug use (Wilcoxon rank sum test, p = 0.51) and between participants with and without drug abuse (Wilcoxon rank sum test, p = 1.0) (**B**) The *Streptococcus* abundance change was not dependent on the duration of PrEP (Spearman’s correlation *ρ* = 0.33 and p = 0.42). *Streptococcus* change and age were not statistically correlated in the eight subjects (*ρ* = 0.33 and p = 0.43). *Streptococcus* abundance change was not sensitive to race (Kruskal-Wallis test, p = 0.11) and was not correlated with the tenofovir diphosphate level at 48 weeks after PrEP (*ρ* = 0.21 and p = 0.62). The *Streptococcus* abundance change was not affected by any drug use (p = 0.64) or drug abuse (p = 0.39).

We next examined the associations between host factors and microbiome abundance changes. Figure 3A plots *Erysipelotrichaceae* abundance difference before and after PrEP as a function of PrEP duration, age, race, TFV-DP level, any drug use status and drug abuse status. The *Erysipelotrichaceae* change was not dependent on either PrEP duration (Spearman’s correlation *ρ* = 0.21 and p = 0.62) or age (*ρ* = 0.071 and p = 0.88). The abundance difference was not correlated with TFV-DP levels at 48 weeks after PrEP (*ρ* = −0.19 and p = 0.66). The *Erysipelotrichaceae* abundance difference was not sensitive to race (Kruskal-Wallis test, p = 0.37) and was not affected by any drug use (Wilcoxon rank sum test, p = 0.51) or drug abuse (Wilcoxon rank sum test, p = 1.0). Likewise, the *Streptococcus* change was not correlated with PrEP duration (*ρ* = 0.33 and p = 0.42). As shown in Fig. 3B, age was not associated with abundance change (*ρ* = 0.33 and p = 0.43). The abundance change was not sensitive to race (Kruskal-Wallis test, p = 0.11) and was not correlated with TFV-DP levels at 48 weeks after PrEP (*ρ* = 0.21 and p = 0.62). The *Streptococcus* abundance difference was not affected by any drug use (p = 0.64) or drug abuse (p = 0.39). This lack of significance of the differences might be due to the small sample size, however, Fig. 3 does not indicate any trends in the associations.

**Figure 4:**
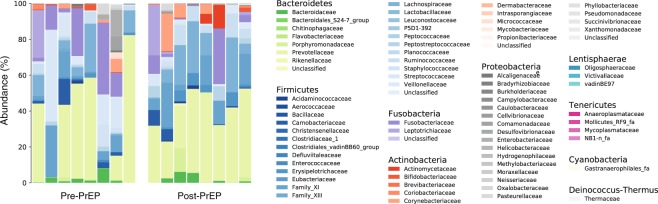
The microbial family composition in eight individuals before and after TDF-FTC PrEP. Subjects are displayed in the pre-PrEP and post-PrEP panels from left to right as follows: WWS, XNT, WD5, WT9, XRQ, 7RJ, 5FX, and 7NR. The phyla *Bacteroidetes* and *Firmicutes* were dominant in all 16 specimens. *Prevotellaceae* was the most abundant family in all specimens except three pre-PrEP specimens (XNT, 7FX, and 7RJ), with average abundance of 36%. *Streptococcaceae* was the most abundant family in pre-PrEP sample of participants XNT (51%) and 5FX (17%) and *Fusobacteriaceae* was the most abundant family in participant 7RJ’s pre-PrEP specimen (40%). The average relative abundance of *Lachnospiraceae* (11%), *Fusobacteriaceae* (9.1%), *Family_XI* (8.8%), and *Streptococcaceae* (6.6%) were observed to be greater than five percent across 16 specimens from eight participants.

Figure 4 compares the family composition of eight individuals before and after PrEP. Consistent with previous reports on the healthy human gut microbiome profiles^19,20^, the CCTG 595 cohort was dominated by the phyla *Bacteroidetes* and *Firmicutes*. *Prevotellaceae* was the most dominant family in all specimens, except three pre-PrEP samples (XNT, 5FX, and 7RJ), with average abundance of 36% across all 16 specimens. Before the initiation of PrEP, the most prevalent family in participant XNT and participant 5FX was *Streptococcaceae* with relative abundances of 51% and 17%, respectively. The most prevalent family in participant 7RJ prior to PrEP initiation was *Fusobacteriaceae* with 40% abundance. Across 16 specimens from eight participants, the average abundance of *Lachnospiraceae* (11%), *Fusobacteriaceae* (9.1%), *Family_XI* (8.8%), and *Streptococcaceae* (6.6%) were observed to be greater than five percent.

**Figure 5:**
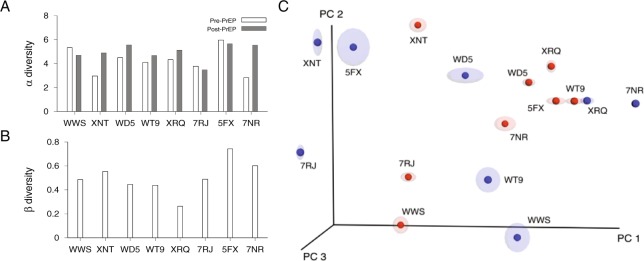
*α* diversity and *β* diversity. (**A**) Within specimen microbial diversity was measured by *α*-diversity (Shannon diversity index). Each participant’s pre-PrEP specimen’s *α* diversity is compared with post-PrEP specimen’s *α* diversity. The *α* diversity did not change significantly with PrEP treatment (Wilcoxon signed-rank test, p = 0.15). (**B**) Microbial *β* diversity was measured between each participant’s pre and post specimens (8 pairs). The weighted UniFrac statistics were computed using QIIME^43^. (**C**) Microbial community variation was represented by principal coordinate analysis of weighted UniFrac distances among specimens. Specimen dissimilarities were projected onto the first three principal coordinate dimensions where pre-PrEP specimens are colored in blue and post-PrEP specimens are in red.

Each participant’s overall microbial diversity was comparable before and after PrEP. Microbiome diversity within a single specimen was assessed by *α* diversity, Shannon diversity index. The dissimilarity between a pair of specimens was assessed by *β* diversity which accounts for phylogenetic tree distances of microbial sequences in a pair of specimens. As shown in Fig. 5A, the average *α* diversity was 4.22 before PrEP and 4.95 after PrEP (Wilcoxon signed-rank test, p = 0.15). We plotted the *β* diversity of each participant’s paired specimens in Fig. 5B. Across 8 pairs, The average *β* diversity across 8 pairs was 0.68 and the paired specimens of the participant XRQ showed the lowest level of *β* diversity. The *β* diversity between each participant’s pre and post-PrEP specimens was smaller than the *β* diversity of pre-PrEP specimen pairs of different individuals (Wilcoxon rank sum test, p = 0.015). Principal coordinate analysis (PCoA) demonstrated that the pre-PrEP and post-PrEP specimens did not form distinct clusters (Fig. 5C). The principal coordinate analysis indicates that there is no global shift in the microbiota between the pre and post PrEP specimens. Individual family and genus abundance changes were inquired through permutation tests in this study, observing that *Erysipelotrichaceae* and *Streptococcus* levels were significantly shifted by PrEP.

**Figure 6:**
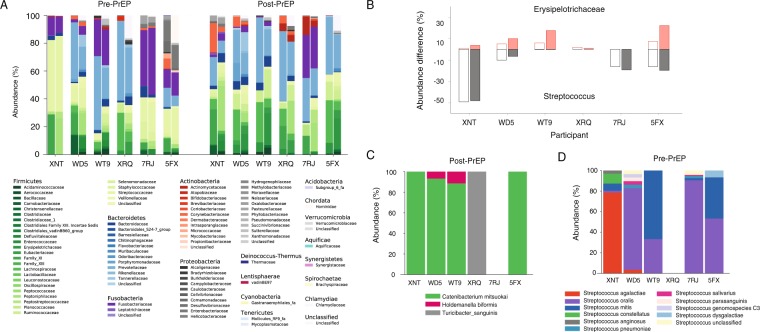
Species identification using full-length 16S-23S sequencing. (**A**) Microbiome Family compositions measured by v4 region of 16s rRNA sequencing (left) and 16S-23S rRNA sequencing (right) for participants, XNT, WD5, WT9, XRQ, 7RJ, and 5FX at pre-PrEP and post-PrEP. (**B**) Abundance differences between pre-PrEP and post-PrEP specimens for *Erysipelotrichaceae* (red) and Streptococcus (grey) for study participants, XNT, WD5, WT9, XRQ, 7RJ, and 5FX. The abundance difference of *Erysipelotrichaceae* measured by 16S-23S sequencing (filled red boxes) was increased in four individuals, compared to the abundance difference obtained from v4-16s sequencing (empty red boxes). The abundance difference of *Streptococcus* measured by 16S-23S sequencing (filled grey boxes) was consistent with that measured by v4-16s sequencing (empty grey boxes). (**C**) Species composition of *Erysipelotrichaceae* family measured from participants XNT, WD5, WT9, XRQ, 7RJ, and 5FX after a minimum of 48 weeks PrEP. Within subjects XNT, WD5, WT9, and 5FX, *Catenibacterium mitsuokai* was the most dominant species in *Erysipelotrichaceae* family. *Turicibacter sanguinis* was detected in participant XRQ and *Holdemanella biformis* was detected in WD5 and WT9. (**D**) Species composition of *Streptococcus* genus measured from the above study participants before PrEP initiation. *Streptococcus agalactiae* (red) was most abundant in participant XNT, *Streptococcus oralis* (purple) was dominant in WD5, 7RJ and 5FX, and *Streptococcus mitis* (blue) was dominant in WT9.

Next we performed full-length 16S-23S rRNA long read sequencing to identify which species in *Erysipelotrichaceae* family and *Streptococcus* genus were affected by long-term PrEP. Figure 2 showed that participants XNT, WD5, WT9, XRQ, 7RJ, 5FX had changes in the abundance of either *Erysipelotrichaceae* or *Streptococcus* and from these participants, we obtained a total of 3363 reads of the 16S-23S rRNA region for species identification (Supplementary Table S2). Figure 6A compares the family composition of pre-PrEP and post-PrEP specimens for both v4–16S short read sequencing and 16S-23S long read sequencing. The overall family profiles were similar; the family composition root-mean-square-error between v4-16S and 16S-23S sequencings ranged from 0.016 to 0.051 across each of 12 specimens from participants XNT, WD5, WT9, XRQ, 7RJ, 5FX. Observable differences in the microbiome profiles for the two sequencing methods were also present (Fig. 6A). We compared abundance changes in *Erysipelotrichaceae* and *Streptococcus* measured by v4-16S sequencing and 16S-23S long read sequencings (Fig. 6B). Post-PrEP *Erysipelotrichaceae* increase measured by 16S-23S sequencing was more prominent than that by v4-16S sequencing. *Streptococcus* decrease measured by 16S-23S sequencing was consistent with that measured by v4-16S sequencing. *Erysipelotrichaceae* increase after PrEP was mainly driven by the species *Catenibacterium mitsuokai*. *Holdemanella biformis* and *Turicibacter sanguinis* were also detected within this family (Fig. 6C). Before PrEP initiation, *Streptococcus agalactiae*, *Streptococcus oralis*, and *Streptococcus mitis* were the dominant species in the *Streptococcus* genus (Fig. 6D).

## Discussion

PrEP use has increased substantially since 2012 and characterizing its long-term side effects is the first step to improve PrEP regimen and adherence^1^. Two well-known side effects of TDF are kidney function decline and bone mineral density decrease^5,6^. Recent studies reported that long-term PrEP users showed impaired kidney function^3^ and a decrease in bone mineral density^4^. The human gut microbiota is known to be correlated with both chronic kidney disease and osteoporosis. Increases in uremic toxins and inflammation among chronic kidney disease patients were in part due to the gut microbiome’s role in the fermentation of short-chain fatty acids, which have an anti-inflammatory impact^21^. Intestinal bacterial overgrowth was associated with changes in essential metabolites for bone processes such as calcium, vitamin K, and vitamin B^12^. During a state of gut microbiome dysbiosis, gut microbiota can affect the absorption of calcium and vitamin D, which can lead to osteoporosis^22^. Our study profiled microbiome structures before and after long-term PrEP to identify microbiota signatures altered by TDF-FTC daily use, which have the potential to affect the kidney and bone health.

We observed that *Streptococcus* was significantly reduced from 12.0% to 1.2% and the *Erysipelotrichaceae* was significantly increased from 0.79% to 3.3% after a minimum of 48 weeks of daily PrEP among CCTG-595 study participants. This cohort is at high risk of HIV-1 infection and has extensive demographic and clinical details including PrEP adherence data. Both *Streptococcus* and *Erysipelotrichaceae* abundance changes were not associated with host factors including PrEP duration, age, race, TFV-DP level, any drug use status and drug abuse status, suggesting that the observed microbiome shifts were potentially induced by daily TDF-FTC use.

Both commensal and pathogenic species of *Streptococcus* have been found to coexist in the healthy human gut microbiome^23^. Members of the *Streptococcus* genus have gut-protective influences. *Streptococcus lactis*, *Streptococcus cremoris*, and *Streptococcus thermophilus* are commonly found in both natural and commensal probiotics^24,25^. Our 16S–23S long read sequencing revealed that *Streptococcus agalactiae*, *Streptococcus oralis*, and *Streptococcus mitis* were the closest species significantly greater in pre-PrEP specimens compared to post-PrEP specimens. *Streptococcus agalactiae* has been reported to be a harmless commensal bacterium, colonized in the intestine^26^. *Streptococcus oralis* and *Streptococcus mitis* are both commensal bacterial which predominately reside in the oral cavity^27^ but these species were also found in fecal samples^28^.

A common enteric side effect associated with TDF-FTC PrEP is a frequently cited reason for poor adherence to PrEP schedules^29^. However, this adverse effect, termed “PrEP start up syndrome”, is usually observed within the first month of TDF-FTC PrEP and resolves spontaneously by 3 months of treatment^30,31^. However, our post-PrEP specimens were collected 48–72 weeks after PrEP initiation. Therefore, a direct association between the enteric side effects and *Streptococcus* reduction should be further investigated at the time of PrEP start up syndrome. Additionally, the observed microbiota differences found using rectal swab specimens may not be comparable to those from other specimen types, collected from different gastrointestinal sites using mucosal biopsies^32,33^.

TDF-FTC also appeared to significantly increase the level of *Erysipelotrichaceae*, a family of bacteria that many species are considered opportunistic pathogens. *Erysipelotrichaceae* has been studied in conjunction with colorectal cancer and metabolic disorders^34^. *Erysipelotrichaceae* was significantly enriched in patients with colorectal cancer, compared to healthy controls^35^. Similarly, an increase in *Erysipelotrichaceae* abundance was reported in colorectal tumor-bearing mice^36^. Additionally, *Erysipelotrichaceae* was increased in obese individuals^37^. Our long read sequencing showed that *Catenibacterium mitsuokai*, *Holdemanella biformis* and *Turicibacter sanguinis* were bacterial species increased after long-term PrEP within *Erysipelotrichaceae* family. Both *Catenibacterium mitsuokai* and *Holdemanella biformis* were associated with gut microbiome dysbiosis caused by high fat and high sugar diet^38^ and an unhealthy fasting lipid profile^39^. Collectively, we speculate that the observed increase in *Erysipelotrichaceae* in the long-term PrEP treated cohort has a potential to contribute to an increased the risk of colorectal cancer and metabolic disorders, while this increase was due to other factors including dietary factors. This speculation is worth further investigation with long-term follow-up of PrEP treated individuals.

In conclusion we observed that *Streptococcus* were decreased and *Erysipelotrichaceae* were increased following daily TDF-FTC for PrEP. Because this study is limited by the small population size, these microbiome shifts should be further examined in a large cohort in conjunction with long-term outcomes. Identifying PrEP microbiota signatures is an important step toward treating and preventing side effects, which is likely to improve PrEP adherence, and thus HIV-1 prevention.

## Methods

### Study participants

Self-collected rectal swab specimens from eight individuals at baseline (pre-PreP) and after taking TDF-FTC daily for 48 to 72 weeks in the California Collaborative Treatment Group (CCTG) study 595 (NCT01761643) were examined^18^. Participants were given a tube with a sterile Dacron rectal swab, along with safety precautions and instructions for rectal swab self-collection. The rectal swab was immediately placed in a sterile 15 ml plain conical tube without media, and the screw cap was secured. The tube containing the sample was transported to the laboratory at room temperature and frozen at −80 °C within two hours of collection. CCTG 595 was a randomized controlled trial in high risk MSM and transgender women to compare the impact of daily text messaging to standard care on adherence to daily TDF-FTC^18^. Regimen adherence was assessed at 48 weeks by testing dried blood spots for tenofovir diphosphate (TFV-DP). This study included only participants with intracellular TFV-DP levels greater than 719 fmol/punch at week 48 weeks, a level associated with taking four or more doses of TDF a week^40^.

Alcohol and substance use were assessed using the SCID substance use screening questionnaire, DAST-10, and AUDIT questionnaires. None of the participants reported diarrhea. The participants’ dietary intake information is not available, which can be an important factor for microbial composition. Six of eight participants reported rectal douching at baseline, one participant reported no use of rectal douching, and one participant declined to answer. All participants signed an informed consent document subject to approval at each site’s IRB (University of Southern California, University of California, San Diego, and Harbor-University of California, Los Angeles). All the procedures and experiments were conducted in accordance with relevant guidelines and regulations.

### Microbiome DNA extraction

The previously frozen swabs were placed in a sterile 1.5 ml Eppendorf tube and the excess swab handle was removed using sterile razor blades or scissors. Microbiome DNA was extracted using the Omega Mag-Bind Universal Pathogen DNA Kit (OMEGA bio-tek). Glass beads, 700 µL of SLX-Mlus Buffer were added to the thawed swab specimen. After 1 minute of vortexing, the tube was centrifuged at 1500 g for 5 minutes. 250 µL of the supernatant was collected, 275 µL SLX-Mlus Buffer was added, and heated to 65 °C for 10 minutes and 95 °C for 10 minutes. Specimens were vortexed at maximum speed using a horizontal adapter (Qiagen) for 8 minutes. After the elution, specimens were purified and concentrated using a Clean & Concentrator-5 kit (Zymo Research). The sample concentration was measured using PicoGreen (Invitrogen).

### 16S ribosomal RNA sequencing

The next-generation sequencing library of the V4 region of the 16S rRNA gene was constructed by the following procedures. The extracted microbiome DNA was amplified using primers 515F-over (5′-TCGTCGGCAGCGTCAGATGTGTATAAGAGACAGGTGCCAGCMGCCGCGGTAA-3′) and 805R-over (5′- GTCTCGTGGGCTCGGAGATGTGTATAAGAGACAGGACTACHVGGGTATCTAATCC-3′) in a reaction volume of 20 µL (5 µL of 0.2 ng/ µL extracted DNA, 13 µL AccuStart II PCR ToughMix, 0.25 µL of 20 µM 515F-over, 0.25 µL of 20 µM 805R-over and 1.5 µL nuclease-free water). The PCR condition was at 94 °C for 3 minutes, followed by 35 cycles of 94 °C for 30 seconds, 60 °C for 30 seconds, then 70 °C for 90 seconds with a final extension at 70 °C for 10 minutes. The PCR product was purified using Agencourt AMPure XP (Beckman Coulter). The index PCR was then performed using primers S5xx (5′-AATGATACGGCGACCACCGAGATCTACACxxxxxxxxTCGTCGGCAGCGTC-3′) and N7xx (5′-CAAGCAGAAGACGGCATACGAGATxxxxxxxxGTCTCGTGGGCTCGG-3′) in a reaction volume of 50 µL (4 µL of purified PCR product, 25 µL AccuStart II PCR ToughMix, 16 µL nuclease-free water, 2.5 µL of 4 µM S5xx and 2.5 µL of 4 µM N7xx). The PCR condition was at 94 °C for 3 minutes, followed by 8 cycles of 94 °C for 30 seconds, 45 °C for 30 seconds, 70 °C for 120 seconds and 10 cycles of 94 °C for 30 seconds, 60 °C for 30 seconds, 70 °C for 120 seconds with a final extension at 70 °C for 10 minutes. The PCR product was purified using Agencourt AMPure Xp (Beckman Coulter). Each sample concentration was determined using PicoGreen (Invitrogen), normalized and pooled and sent to the University of California, Los Angeles Technology Center for Genomics and Bioinformatics for Illumina MiSeq sequencing.

### 16S–23S ribosomal RNA sequencing

We performed 16S-23S rRNA long read sequencing on pre-PrEP and post-PrEP specimens from participants XNT, WD5, WT9, XRQ, 7RJ, 5FX. Additionally, a microbiome standard (ATCC, MSA-1001) consisting of 10 control strains was sequenced. Each participant’s extracted microbiome DNA (as described above) was amplified using primers 27F (5′-AGAGTTTGATCMTGGCTCAG-3′) and 2409R23 (5′-GACATCGAGGTGCCAAAC-3′), targeting around 4500 base long 16S-23S rRNA region. PCR was performed in a reaction volume of 20 µL (5 µL of 0.2 ng/ µL extracted DNA, 13 µL of 2x AccuStart II PCR ToughMix, 1.5 µL nuclease-free water, 0.25 µL of 20 µM 27 F and 0.25 µL of 20 µM 2409R23). The PCR condition was 94 °C for 3 minutes, 35 cycles of 94 °C for 30 seconds, 60 °C for 30 seconds, 70 °C for 5 minutes, and a final extension at 70 °C for 10 minutes. The PCR product was purified with 1.8x Ampure XP Beads, washed twice with 70% ethanol, and eluted in 20 µL of nuclease free water.

The purified samples were then set up for an index PCR to barcode each sample using primers, 27Fxx (5′-xxxxxxxxxxxxAGAGTTTGATCMTGGCTCAG-3′) and 2409R23xx (5′-xxxxxxxxxxxxGACATCGAGGTGCCAAAC-3′). The index PCR was performed in a 50 µL reaction volume with 5 µL of purified PCR product, 25 µL AccuStart II PCR ToughMix, 17.6 µL nuclease-free water, 1.2 µL of 4 µM 27Fxx and 1.2 µL of 4 µM 2409R23xx. The PCR condition was at 94 °C for 3 minutes, followed by 10 cycles of 94 °C for 30 seconds, 63 °C for 20 seconds, 70 °C for 5 minutes, and a final extension at 70 C for 10 minutes. The index PCR condition for specimen pre-PrEP 5FX was modified by increasing primer volumes (2.5 µL), increasing the purified PCR product volume (7 µL), lowering the annealing temperature (60 °C), and increasing annealing time (60 °C for 30 seconds).

A PicoGreen assay was performed to determine the concentration of each sample. Equimolar amounts of each of 13 samples were pooled and shipped overnight on dry ice to RTL genomics for PacBio sequencing. After arrival, the samples were checked for concentration using the dsDNA Broad Range DNA kit on the Qubit Fluorometer 3.0 and quality checked with a Fragment Analyzer by Advanced Analytical Technologies using the High Sensitivity Large Fragment 50KB Analysis kit, which were subject to SMRTbell library preparation for PacBio Sequencing. This process began with a 0.5x Ampure PB Purification A total of 600 ng of DNA was used for creating circularized double stranded DNA templates by ligating hairpin adaptors to each side of templates. Following the overnight ligation, the circular templates were purified with AMPure PB Beads and SMRTbell sequencing primers were annealed to the adaptors. The dsDNA templates were then prepared for loading with a final concentration of 6 pM to initiate SMRTbell template sequencing.

### Sequence data analysis

The forward and reverse reads obtained from the MiSeq sequencing run were assembled using the command, *make*.*contigs* in MOTHUR^41^. The obtained fasta file was screened for the presence of ambiguous bases and any reads with such bases were discarded. The reads were then subjected to taxonomy annotation after putative chimeric sequences were removed using *chimera*.*uchime*. The Silva full length sequences and taxonomy references, consisting of 190,661 sequences (release 128), were used for taxonomy annotation^42^. To expedite taxonomy annotation, clustering was performed using *pre*.*cluster* by grouping reads that are within one mismatch of each other. Supplementary Table S1 shows the number of total reads and unique reads, prior to processing and post processing for each of the 16 specimens sequenced in this study.

Each read obtained from a 16S-23S rRNA sequencing run was subjected to taxonomy annotation after removing putative chimeric sequences and clustering. BLAST with NCBI nucleotide collection database was used to find the bacterial species with the best match for each read. When the best matched strain showed less than 90% identity or the length of the best matched strain was less than 25% of each read length, the read was labeled as unclassified.

Each specimen’s *α* diversity with the Shannon diversity index was measured using *alpha_diversity*.*py* in QIIME^43^. To calculate *β* diversity, all 16 specimens’ fasta files were combined as a single file. Closely related sequences with similarities greater than 97% were grouped as an operational taxonomic unit (OTU) using *pick_otus*.*py* in QIIME and each OTU’s representative sequence was picked using *pick_rep_set*.*py*. The selected representative sequences were aligned using *align_seqs*.*py* with the MUSCLE algorithm. Then the OTU table and phylogenetic tree were obtained using *make_otu_table*.*py* and *make_phylogeny*.*py*, respectively. Using QIIME *jackknifed_beta_diversity*.*py*, jackknife resampling was performed by sampling 1% of the total sequences and repeated 10 times to obtain specimen pair’s *β* diversity matrices and principal coordinate analysis (PCoA) plots.

### Permutation t test

We statistically examined differences in family or genus abundance between pre-PrEP and post-PrEP specimens. The standard t-tests for paired measurements assume independence, normality, and constant variance. With abundance proportions, it is likely that normality and constant variance are not held and the sample size is not large enough to use approximate normality of the mean. Instead, we conducted a permutation test in order to empirically assess the asymptotic p-values. The permutation test has been widely recommended in multiple statistical testing^44–46^. For each family or genus, a *t*-statistic was computed on the observed diversity data as follows:

1 $$t=\frac{POS{T}_{av}-PR{E}_{av}}{\sqrt{(POS{T}_{var}+Pr{e}_{var})/n}},$$

where *n* is the number of participants, *POST*_*av*_ (*PRE*_*av*_) is the average abundance of a given family or genus among post-PrEP (pre-PrEP) specimens, and *POST*_*var*_ (*PRE*_*var*_) is the abundance variance of a given family or genus among post-PrEP (pre-PrEP) specimens. The PrEP status was then randomly permuted, holding the numbers of pre-PrEP and post-PrEP specimens constant over permutations; a total of 100,000 such permutations were completed. A *t*-statistic in Eq. (1) was computed for each permutation and the empirical p-value of each family or genus was calculated as the proportion of permutations with a *t*-test statistic greater than or equal to the *t*-test statistic derived from the actual data when the original *t* value was greater than zero. Otherwise, the empiric p-value was determined as the proportion of permutations with a *t*-test statistic less than or equal to the negative original *t* value. Based on this empirical p-value, the families or genera with significant differences in abundance between pre-PrEP and post-PrEP were determined. We also performed paired permutation tests for a given family or genus where we permuted each participant’s pre-PrEP and post-PrEP abundance values, yielding a total of 2^*n*^ permutations. For each permutation, a t-statistic was measured and the empirical p-value was obtained from the proportion of permuted t-statistics that were more extreme than the observed data t-statistic.

## Electronic supplementary material

Supplementary Information

## Data Availability

All microbiome sequence data from this study are available at EBI with accession numbers PRJEB29018 and PRJEB29019.
