# Supplementary material for: Daily HIV pre-exposure prophylaxis (PrEP) with tenofovir disoproxil fumarate-emtricitabine reduced Streptococcus and increased Erysipelotrichaceae in rectal microbiota
Source: Sci Rep. 2018 Oct 12;8:15212. doi: 10.1038/s41598-018-33524-6 (PMC6185988; doi:10.1038/s41598-018-33524-6)
Supplement: Supplementary file 1 — Supplementary Information [file 41598_2018_33524_MOESM1_ESM.docx]

Supplementary Information

Daily HIV pre-exposure prophylaxis (PrEP) with tenofovir disoproxil fumarate-emtricitabine reduced *Streptococcus* and increased *Erysipelotrichaceae* in rectal microbiota

Michael P. Dubé^1^, Sung Yong Park^2^, Heather Ross^2^, Tanzy M. T. Love^3^, Sheldon R. Morris^4^, and Ha Youn Lee^2*^

^1^Department of Medicine and Division of Infectious Diseases, Keck School of Medicine, University of Southern California, Los Angeles, CA.

^2^Department of Molecular Microbiology and Immunology, Keck School of Medicine, University of Southern California, Los Angeles, CA.

^3^Department of Biostatistics and Computational Biology, School of Medicine and Dentistry, University of Rochester, Rochester, NY.

^4^University of California San Diego Antiviral Research Center, San Diego, CA.

^*^e-mail: hayoun@usc.edu

|  | | Pre-Processing | | Post-Processing | |
| --- | --- | --- | --- | --- | --- |
| Participant | | Number  of Total Reads | Number  of Unique Reads | Number  of Total Reads | Number  of Grouped Reads |
| Pre-PrEP | WWS | 138,792 | 72,097 | 112,916 | 18,746 |
|  | XNT | 126,059 | 65,532 | 93,500 | 14,222 |
|  | WD5 | 134,398 | 69,508 | 103,793 | 14,462 |
|  | WT9 | 131,826 | 65,126 | 106,931 | 15,287 |
|  | XRQ | 137,004 | 62,583 | 114,407 | 15,634 |
|  | 7RJ | 149,231 | 74,923 | 115,892 | 18,067 |
|  | 5FX | 147,189 | 107,982 | 38,957 | 9,093 |
|  | 7NR | 130,854 | 59,144 | 110,066 | 14,924 |
| Post-Prep | WWS | 129,772 | 63,253 | 103,743 | 15,291 |
|  | XNT | 119,640 | 61,671 | 95,650 | 14,518 |
|  | WD5 | 124,472 | 68,734 | 94,516 | 13,903 |
|  | WT9 | 117,189 | 54,799 | 97,001 | 13,299 |
|  | XRQ | 129,215 | 62,054 | 106,616 | 14,411 |
|  | 7RJ | 110,037 | 50,723 | 89,651 | 10,948 |
|  | 5FX | 129,497 | 67,688 | 105,179 | 16,564 |
|  | 7NR | 126,448 | 68,360 | 100,686 | 16,042 |
| Mean | | 130,101 | 67,136 | 99,344 | 14,713 |
| Median | | 129,635 | 65,329 | 103,768 | 14,721 |
| Standard Deviation | | 10,147 | 12,509 | 17,893 | 2,354 |

**Table S1.** Number of reads and unique reads prior to processing and number of reads and uniquely grouped reads after processing.

|  | Pre-PrEP | Post-PrEP |
| --- | --- | --- |
| Participant | Number  of Total Reads | Number  of Total Reads |
| XNT  WD5  WT9  XRQ  7RJ  5FX | 426 | 320 |
|  | 209 | 274 |
|  | 338 | 423 |
|  | 173 | 106 |
|  | 446 | 344 |
|  | 66 | 238 |
| Mean | 276.3 | 284.2 |
| Median | 273.5 | 297 |
| Standard Deviation | 151.3 | 107.8 |

**Table S2.** Number of 16s-23s reads obtained from each participant’s pre-PrEP and post-PrEP specimens.
